# Somatic non-cancerous overgrowth syndrome of obscure molecular etiology: what are the causes and options?

**DOI:** 10.1007/s00109-022-02214-2

**Published:** 2022-06-03

**Authors:** Alexandre P. Garneau, Ludwig Haydock, Laurence E. Tremblay, Pierre-Luc Harvey-Michaud, Yun-Hua Esther Hsiao, Samuel P. Strom, Guillaume Canaud, Paul Isenring

**Affiliations:** 1 Nephrology Research Group, Department of Medicine, Faculty of Medicine, L’Hôtel-Dieu de Québec du CHU de Québec, Laval University, Quebec City, Québec G1R 2J6 Canada; 2 Fulgent Genetics, Temple City, CA 91780 USA; 3 Unité d’hypercroissance dysharmonieuse, Hôpital Necker-Enfants Malades, AP-HP, Inserm U1151, Université de Paris, rue de Sèvres, 75105 Paris, France

Somatic overgrowth syndromes (SOSs) are most commonly due to an embryonic mutation in a regulator, member or effector of the PIK3CA/AKT/mTOR pathway [1]. They can affect any tissues in the body but are usually segmental in presentation and cause significant physical, and psychological morbidity. Their management has been traditionally supportive consisting of invasive debulking surgeries, vascular embolizations, physical therapy and psychological assistance.

When the gene at cause is *PIK3CA* 1 itself, as is most frequently the case, the SOS is then referred to as PIK3CA-related overgrowth syndrome (PROS) but is still associated with diverse developmental phenotypes such as isolated digital hypertrophy, generalized fibroadipose overgrowth, and CLOVES syndrome^2^ [2–5]. Some of these phenotypes are also seen in association with mutations in other proliferation/differentiation genes.

A molecular diagnosis should be obtained in all cases of SOSs to determine whether a pharmacological treatment could be indicated [1–6]. One such treatment consists of the oral PIK3CA inhibitor alpelisib^3^ [7] that has shown efficacy in the treatment of PROS based on case reports, animal models, and ongoing clinical studies [1–5]. However, it has been offered thus far almost exclusively to patients in whom somatic DNA testing showed SOS to result from a gain-of-function mutation in the target enzyme.

One must nonetheless remember that the underlying somatic defect in SOS escapes identification in over 30% of patients [1–5, 8], that *PIK3CA* is often the gene involved and that life-threatening complications can occur at any time. Unfortunately, the underlying molecular defect cannot be deduced with certainty from the clinical presentation alone given that there is much overlap in disease expression among the various types of SOSs.

An important question that thus comes to mind is whether PIK3CA inhibition could be worth the try when the etiology of SOS in an affected patient could not be categorized based on somatic DNA testing. We are taking the opportunity of the current commentary to illustrate our point of view in this regard and are doing so through a case of SOS that we have recently investigated and managed through precision medicine.

The case at issue is that of a 23-year-old woman who had experienced a life-long history of right-sided hemihypertrophy with vascular malformations in many organs. Her past medical history was otherwise unremarkable, except for the SOS and complications that this condition had led to (described below). As for the medication history, it consisted essentially of oral iron supplements on an irregular basis.

During our initial evaluation (Fig. 1), we observed that the right-sided hemihypertrophy was caused by adipose overgrowth in the extremities as well as superficial and deep-seated venous ectasias throughout the limbs. An epidermal nevus was also present near the elbow, a large venous mass along the vaginal upper outer lip and numerous venous ectasias in the bladder, uterus, and colon (Fig. 1). Many of these manifestations had progressed up to the end of teenagerhood.

**Fig. 1:**
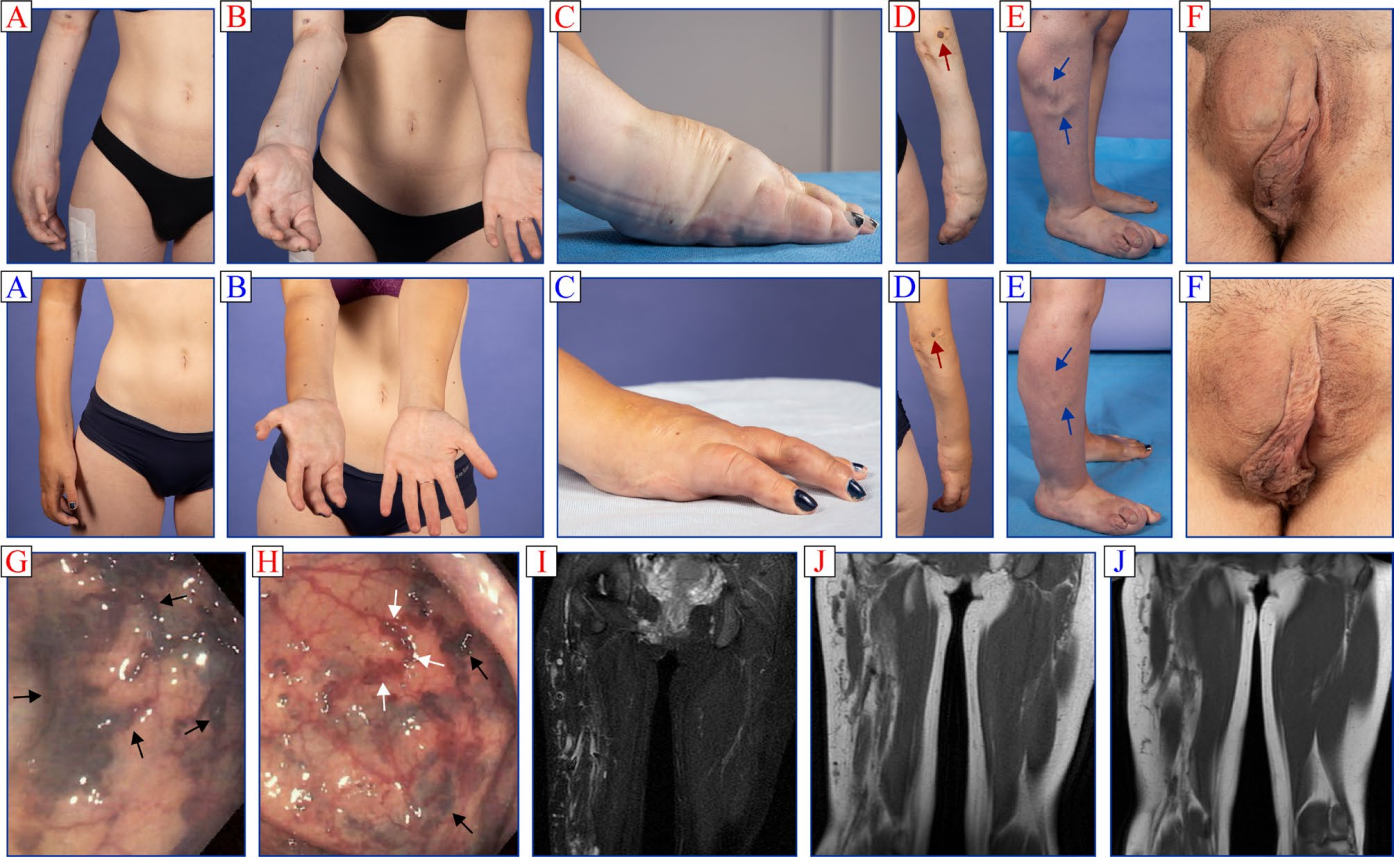
Clinical manifestations at baseline (**A** to **F** and **G** to **J** in red) and after 2 months on alpelisib (**A** to **F** and **J** in blue). **A**–**F** in red, external appearance before treatment. Right distal arm is seen to be severely enlarged (panels **A** to **D**) with an epidermal nevus near the elbow (red arrows in panels **D**), right distal leg to be moderately enlarged (panel **E**) with a large venous ectasia (blue arrows in the same panels), and upper outer lip of vagina to harbor a large venous mass (panels **F**). Mild hypertrichosis was also present in the distal hypertrophied extremities (barely visible on the pictures shown). **A**–**F** in blue, external appearance during treatment. Areas of overgrowth and epidermal nevus have decreased by more than 50% (panels **A** to **E**) and so has hypertrichosis (barely visible on the pictures shown), while the venous ectasia and perineal mass have almost completely disappeared (panels **E** and **F**). **G** to **J** in red internal vascular malformations before treatment. Appearance of sigmoid (panel **G**) and colon at the splenic angle (panel **H**) based on endoscopic images. The mucosae are seen to harbor multiple vascular ectasias (black arrows in panels **G** and **H**) and vascular tortuosities (white arrows in panel **H**). Appearance of lower body based on MRI STIR- or T1-weighted images (panels **I** and **J**). Right leg, uterus and bladder are all seen to harbor multiple venous malformations. Note that the right hand and right foot were both affected by moderate adipose overgrowth (not shown). **J** in blue internal vascular malformations during treatment. Vascular malformations have decreased by more than 60% based on MRI T1-weighted images. A control colonoscopy has still to be conducted

In regard to the complications associated with SOS, they were as follows: (1) chronic right-sided pain especially in the arm, (2) chronic iron-deficiency anemia due to recurrent episodes of macroscopic hematuria, moderate-to-severe intestinal blood loss, and menorrhagia, and (3) bone deformities such as a right knee valgus, scoliosis of the spine, and deviation of the right little finger.

Between ages 3 and 15, the SOS had been managed through excision of the little finger, of the second and third toes, of epidermal nevi, and superficial venous masses. These interventions had been carried out to facilitate clothing, maximize usage of extremities, alleviate discomfort or bruising associated with some of the lesions, and improve aesthetic appearance. The patient had also been considered for pelvic embolization or hysterectomy because of the blood losses.

As for the underlying genetic defect, it was searched for during our initial evaluation in tissue samples derived from two different sites of overgrowth. High-coverage (> 2000 ×) next-generation targeted exome sequencing and deletion/duplication analysis were carried out with a lower limit frequency filter of 2.0% or less to analyze *PIK3CA* in one sample and 171 cancer-related genes^4^ in the other. Low-coverage (20 ×) whole genome sequencing was also carried out to analyze the DNA of one sample. A number of variants were identified, but none was considered pathogenic.^5^

The patient was started on alpelisib (at a maintenance dose of 250 mg/day) even though DNA testing was inconclusive and without prior in vitro studies to test for potential efficacy [9].^6^ To our surprise, and at the expense of minimal side effects such as diarrhea and oral ulcers in the first few weeks, pain in the right arm completely subsided after less than 14 days, while adipose overgrowth and vascular malformations decreased by 50 to 80% after 5 months (see Fig. 1) with no further episodes of blood externalization.

The current case description is thus remarkable for illustrating the potential worth of treating SOS pharmacologically even when attempts to uncover the causative gene failed. PIK3CA could be seen as an initial target of choice in this setting because: (1) it is often the gene involved in SOS, (2) its inhibition is well-tolerated and effective in the treatment of PROS [1–5], and (3) SOSs can be associated with upstream regulators of PIKC3A (e.g., TEK, GNAQ, RAS) and improve under alpelisib even then (personal observations).

For the same reasons, it is not intuitive that the mTOR inhibitor rapamycin would constitute a superior or even comparable alternative in the absence of identifiable molecular defects. Although it has been used in the past to treat orphan SOSs as well as PIK3CA-, RAS-, TEK- and GNAQ-associated SOSs with some success [10], it often comes with bothersome adverse effects, and its primary target is downstream of PIK3CA.

It is unclear why the molecular defect in SOS is typically hard to catch. One possibility is that the genetic variant at cause is still considered to be of unknown significance. Alternatively, it could already be known for being of pathogenic significance but is present in the tissue tested at such a low allelic frequency that it is filtered out before data analysis. In this regard, short-length mosaic copy number variations (CNVs) are especially easy to miss by next generation sequencing when they are in a mosaic state.

When a somatic defect is identified in PROS, it is in fact commonly very low in allelic frequency, i.e., close to, or even below 2%. Surprisingly, however, the tissues that are used for DNA testing under such circumstances are generally chosen from grossly affected areas. While this apparent discrepancy between genotype and phenotype could be seen as irrelevant, it might indicate on the contrary that mutated cells in SOS can passage growth factors to neighboring wildtype cells and exert a strong dilution effect on mosaicism as a consequence.

Of notice, pain in the areas of affected tissues is a common manifestation of PROS [3]. It has also been found in many individuals to decrease rapidly (often in a matter of days) following PIK3CA inhibition, that is, well before overgrowth could have regressed substantially. It would thus appear that an overactive PIK3CA in these tissues causes them to become painful by stimulating the activity of nociceptive intermediates in the central or peripheral nervous system [3, 11].

There are probably many other patients like ours who could benefit from a targeted therapy but are denied such as chance. This impression is supported by the very low number of individuals who are currently receiving alpelisib in Canada for an SOS. We have now entered an era of precision medicine where previously intractable deforming diseases can begin to be cured. This new era will probably come to be known as that of the *pharmacological scalpels*.

## Data Availability

Data will be made fully available upon request.

## Footnotes

1 PIK3CA is the p110α subunit of phosphatidylinositol 3-kinase and a member of the PIK3CA/AKT/mTOR transduction pathway. Over 40 different mutations have been identified in PROS [1, 4].

2 CLOVES is a prototypal form of PROS.

3 Alpelisib (Novartis) is a drug that was developed for the treatment of PIK3CA-mutated, hormone receptor-positive advanced breast cancer [7].

4 The exome panel (TruSight Tumor 170 test, Illumina Inc.) included more specifically the following genes: *ABL1*, *AKT1*, *AKT2*, *AKT3*, *ALK*, *APC*, *AR*, *ARID1A*, *ATM*, *ATR*, *AXL*, *BAP1*, *BARD1*, *BCL2*, *BCL6*, *BRAF*, *BRCA1*, *BRCA2*, *BRIP1*, *BTK*, *CARD11*, *CCND1*, *CCND2*, *CCND3*, *CCNE1*, *CD79A*, *CD79B*, *CDH1*, *CDK12*, *CDK4*, *CDK6*, *CDKN2A*, *CEBPA*, *CHEK1*, *CHEK2*, *CREBBP*, *CSF1R*, *CTNNB1*, *DDR2*, *DNMT3A*, *EGFR*, *EML4*, *EP300*, *ERBB2*, *ERBB3*, *ERBB4*, *ERCC1*, *ERCC2*, *ERG*, *ESR1*, *ETS1*, *ETV1*, *ETV4*, *ETV5*, *EWSR1*, *EZH2*, *FAM175A*, *FANCI*, *FANCL*, *FBXW7*, *FGF1*, *FGF10*, *FGF14*, *FGF19*, *FGF2*, *FGF23*, *FGF3*, *FGF4*, *FGF5*, *FGF6*, *FGF7*, *FGF8*, *FGF9*, *FGFR1*, *FGFR2*, *FGFR3*, *FGFR4*, *FLI1*, *FLT1*, *FLT3*, *FOXL2*, *GEN1*, *GNA11*, *GNAQ*, *GNAS*, *HNF1A*, *HRAS*, *IDH1*, *IDH2*, *INPP4B*, *JAK2*, *JAK3*, *KDR*, *KIF5B*, *KIT*, *KMT2A*, *KRAS*, *LAMP1*, *MAP2K1*, *MAP2K2*, *MCL1*, *MDM2*, *MDM4*, *MET*, *MLH1*, *MLLT3*, *MPL*, *MRE11A*, *MSH2*, *MSH3*, *MSH6*, *MTOR*, *MUTYH*, *MYC*, *MYCL1*, *MYCN*, *MYD88*, *NBN*, *NF1*, *NOTCH1*, *NOTCH2*, *NOTCH3*, *NPM1*, *NRAS*, *NRG1*, *NTRK1*, *NTRK2*, *NTRK3*, *PALB2*, *PAX3*, *PAX7*, *PDGFRA*, *PDGFRB*, *PIK3CA*, *PIK3CB*, *PIK3CD*, *PIK3CG*, *PIK3R1*, *PMS2*, *PPARG*, *PPP2R2A*, *PTCH1*, *PTEN*, *PTPN11*, *RAD51*, *RAD51B*, *RAD51C*, *RAD51D*, *RAD54L*, *RAF1*, *RB1*, *RET*, *RICTOR*, *ROS1*, *RPS6KB1*, *SLX4*, *SMAD4*, *SMARCB1*, *SMO*, *SRC*, *STK11*, *TERT*, *TET2*, *TFRC*, *TMPRSS2*, *TP53*, *TSC1*, *TSC2*, *VHL*, and *XRCC2. TEK* was also included in the analyses at a coverage of over > 2000 ×.

5 The promoter regions of *PIK3CA* and *TEK* also revealed normal.

6 After approval by Health Canada, Novartis provided (and still provides) Alpelisib to this patient on a compassionate and an off-label basis through its Managed Accessed Programs. Its reasons for accepting to do so in the absence of molecular diagnosis and of further in vitro studies [9] were as follows: 1) the phenotype was consistent with PROS, 2) the SOS was associated with debilitating complications and 3) the patient had already been subjected to an exhaustive genetic investigation.

## Abbreviations

AKT: Protein kinase B
*CLOVES*: Congenital lipomatous overgrowth with vascular malformations, epidermal nevi and skeletal abnormalities
GNAQ: G protein subunit αq
mTOR: Mammalian target of rapamycin
PIK3CA: Phosphatidylinositol 3-kinase type C alpha
PROS: PI3KCA-related overgrowth syndrome
RAS: Rat sarcoma virus
SOS: Somatic overgrowth syndrome
TEK: Tyrosine kinase with EGF homology domain
